# Comparing Nighttime to Rush Hour Avoidance as Initial Driving Self-Regulation Behaviors

**DOI:** 10.1093/geroni/igab046.1019

**Published:** 2021-12-17

**Authors:** Jonathon Vivoda, Lisa Molnar, David Eby, Carolyn DiGuiseppi, Vanya Jones, Guohua Li, Thelma Mielenz, David Strogatz

**Affiliations:** 1 Miami University, Oxford, Ohio, United States; 2 University of Michigan Transportation Research Institute, Ann Arbor, Michigan, United States; 3 University of Colorado Denver, Aurora, Colorado, United States; 4 Johns Hopkins University, Baltimore, Maryland, United States; 5 Columbia University, New York, New York, United States; 6 Bassett Research Institute, Cooperstown, New York, United States

## Abstract

Aging is associated with an increase in avoidance of challenging driving situations (e.g., driving at night, during rush hour, on freeways, and in unfamiliar areas). Such avoidance behavior may be due to driving self-regulation (SR), an intentional response to perceived declining abilities, or it may be due to other factors such as lifestyle changes or preferences. Most previous research has not studied SR as the reason for avoidance, and has treated avoidance behaviors interchangeably. In addition, previous research has not differentiated one’s first SR behavior from those reported later in the process. This study included 1,557 participants from the AAA Longitudinal Research on Aging Drivers (LongROAD) to assess older adults’ initial self-regulatory behavior by comparing the frequency of nighttime, rush hour, freeway, and unfamiliar area avoidance among those who reported only one SR behavior. Nighttime SR was most common (58.8%), followed by rush hour (25.5%), unfamiliar areas (11.0%), and freeways (4.8%). Binary logistic regression was used to assess how demographics, function, and self-reported driving variables were related to different odds of reporting nighttime vs. rush hour avoidance (the two most common) as one’s initial SR behavior. Higher odds of reporting nighttime avoidance (compared to rush hour) as one’s initial SR behavior were related to female gender, low income, impaired visual acuity, better self-reported ability to see during the day, worse self-reported ability to see at night, less comfort driving at night, and more comfort driving during rush hour, and in unfamiliar areas.

